# Physical Activity Pattern Characterized by Domains and Dimensions of the Roma Population in Comparison with That of the General Population in Northeast Hungary

**DOI:** 10.3390/ijerph19063545

**Published:** 2022-03-16

**Authors:** Éva Bácsné Bába, Péter Pikó, Anetta Müller, Gergely Ráthonyi, Péter Balogh, Zsigmond Kósa, Nóra Kovács, János Sándor, Róza Ádány, Zoltán Bács

**Affiliations:** 1 Institute of Sport Economics and Management, Faculty of Economics and Business, University of Debrecen, 4032 Debrecen, Hungary; bacsne.baba.eva@econ.unideb.hu (É.B.B.); muller.anetta@econ.unideb.hu (A.M.); 2 MTA-DE Public Health Research Group, University of Debrecen, 4032 Debrecen, Hungary; piko.peter@med.unideb.hu (P.P.); adany.roza@med.unideb.hu (R.Á.); 3 Department of Statistics and Methodology, Faculty of Economics and Business, University of Debrecen, 4032 Debrecen, Hungary; balogh.peter@econ.unideb.hu; 4 Department of Health Methodology and Public Health, Faculty of Health, University of Debrecen, 4400 Nyíregyháza, Hungary; kosa.zsigmond@foh.unideb.hu; 5 Department of Public Health and Epidemiology, Faculty of Medicine, University of Debrecen, 4032 Debrecen, Hungary; kovacs.nora@med.unideb.hu (N.K.); sandor.janos@med.unideb.hu (J.S.); 6 Department of Accounting, Faculty of Economics and Business, University of Debrecen, 4032 Debrecen, Hungary; bacs.zoltan@econ.unideb.hu

**Keywords:** physical activity, International Physical Activity Questionnaire (IPAQ), Roma, Metabolic Equivalent Task minutes per week (MET), physical activity dimensions, physical activity domains

## Abstract

Our study focuses on examining physical activity, as one of the most influential health determinants by domains and dimensions among Roma, the largest vulnerable ethnic minority in Europe. The study was carried out on a sample representative of the Hungarian Roma (HR) population (*n* = 350) living in segregated colonies in Northeast Hungary in comparison with the Hungarian general (HG) population sample (*n* = 343) from the same region. Data were collected using the International Physical Activity Questionnaire (IPAQ) long-form and physical activity was quantified as MET-min/week. Scores were calculated for walking, moderate and vigorous-intensity activities for each domain (work, transport, domestic and gardening, leisure) and as an overall total. The HR population—similarly to the HG—is characterized by moderate or high physical activity; however, this level is achieved by work and housework/gardening instead of leisure time activities, which is worryingly low among HR females, but its prevalence is significantly (*p* < 0.001) lower in both sexes than among the HG population in the vigorous activity category. HR men move (walk and cycle) significantly more during transport than HG men. Our results may direct the attention of decision-makers to improve the health of Roma by increasing leisure-time physical activity.

## 1. Introduction

“Transforming our World: the 2030 Agenda for Sustainable Development” is the first United Nations’ strategic document with a strong focus on disadvantaged members of societies, in harmony with its motto “leave no one behind” [1]. Reflecting on the 2030 Agenda’s goal 3 “Ensure healthy lives and promote wellbeing for all at all ages” and other health-related sustainable development goals, the World Health Organization initiated the Global Action Plan [2] which highlights the need for a strong focus on improving equity and meeting the needs of vulnerable populations. One of the most challenging public health issues today is to reduce inequalities in health and access to health care services. An unfavourable health status is a long-standing problem among the Roma population, which is the largest ethnic minority population with an estimated number of 10–12 million in Europe and concentrating in the Central Eastern European countries, among them in Hungary [3], and who have a worse health status than the majority of the population regardless of the country where they live [4,5,6,7,8]. The causes of the poor health of Roma are multifunctional [3,4], but they are mostly related to their poor socioeconomic status resulting in the high prevalence of risk factors linked to the development of both communicable and non-communicable diseases.

Regarding health behaviour, it is well known that smoking prevalence is very high among Roma [9,10,11,12], and alcohol consumption became significantly more frequent in the younger age groups of both sexes during the decade (2005–2015), dedicated to Roma inclusion [12], and studies from different European countries on their dietary characteristics have revealed a suboptimal dietary profile and nutritional status among the Roma [13,14,15].

In general, Roma People have unhealthy behaviour and are presumed to have lower levels of physical activity, which put them at increased risk of developing metabolic or cardiovascular disorders [16,17]. It is well known that higher levels of physical activity provide substantial benefits for overall health [18], and regular physical activity is associated with a reduction in cardiovascular [19,20,21] and obesity risks [22,23]. Several Roma studies showing an increased risk of cardiometabolic diseases among them regardless of the country in which they live were published previously. Obesity [6,15,24,25,26,27,28], diabetes [6,24,26,29], insulin resistance [27], smoking [5,9,15,24,27,29], physical inactivity [30,31], hypertension [6,24,26], abnormal lipid profile [6,15,26], and metabolic syndrome [6,14,26] have high prevalence, and significantly, they were found to be more common among the Roma.

In our recent study, we have assessed the estimation of a 10-year risk of development of fatal and nonfatal cardiovascular diseases (CVDs) based on the most used risk assessment scoring models and compared it with the risk of the Hungarian general (HG) population. For all CVD risk estimation scores, the average of the estimated risk was higher among Roma compared to the HG independently of sex. The proportion of high-risk group in the Hungarian Roma male population was on average 1.5–3 times higher than in the general one, while among Roma females, the proportion of high-risk groups in the Hungarian Roma female population was on average 2–3 times higher compared to the distribution of females in the general population [32]. By defining the metabolic syndrome (MetS) prevalence by age groups, we could show that MetS arises earlier among the Roma than in the HG population; while there is no significant difference in MetS prevalence between HG and HR populations in the 20–34 years age group (29.6% and 26.4%, respectively), it is significantly higher in the HR population than in the HG population in the 35–49 years age group (46.7% vs. 31.6%), i.e., in comparison with the prevalence values for the age group of 20–34 years, a significant increase in MetS prevalence can be observed in the age group of 35–49 years among Roma, while in case of the HG population, it can be detected only in the older age group (50–64 years) [33].

However, the physical activity of Roma people has hardly been characterized; data on physical activity from the cross-sectional population-based HepaMeta study conducted in Slovakia in 2011 showed a significant difference in leisure-time physical activities which was found to be significantly lower among Roma women than among non-Roma women [34]. Regarding health-endangering behaviours among Slovak Roma adolescents living in segregated colonies in comparison to non-Roma adolescents, differences were statistically not significant, except physical inactivity rates which were significantly higher among Roma girls [35]. Even these sporadic findings are available from studies with methodological deficiencies. Data on physical activity were collected with a single question regarding the frequency of physical activity, and respondents could estimate it on a 4-point Likert scale [35]. The answers were dichotomised into two categories such as “lack” or “frequent” physical activity, and even in the more appropriate HepaMeta study [36], dimensions (mode or type of activity, frequency, and duration of and intensity of performing activity) and domains (leisure-time physical activity, occupational activity, travel, domestic/gardening) of physical activity were not properly considered.

Our research focuses on examining the physical activity of the Roma. We hypothesized that the Roma are characterized by lower physical activity compared to the majority of people in society due to their socioeconomic disadvantages. We hypothesized that their domestic and garden physical activity dominated mainly because of their rural lifestyle, and we expected transport related physical activity to be high due to walking and cycling. Our study is the first assessment of physical activity among Roma using the most accepted instrument, the long form of the International Physical Activity Questionnaire (IPAQ), and calculating activity in Metabolic Equivalent Task minutes per week (MET-min/week) according to the IPAQ scoring protocol [36].

Detailed characterization of physical activity, i.e., describing the physical activity pattern by domains and dimensions of the Roma population in comparison with that of the general population is essential to define gaps and to develop targeted interventions at the community level and in this way to improve health and prevent diseases among Roma people.

## 2. Materials and Methods

### 2.1. Sample Populations

Data used in our present study were obtained in a cross-sectional survey carried out between May and August 2018, as a three-pillar (i.e., questionnaire-based, physical examination, and laboratory examination) complex (i.e., health behaviour and examination) survey. Sampling and data collection are described in detail elsewhere [33]. Briefly, both HR and HG samples were recruited from two counties (Hajdú-Bihar and Szabolcs-Szatmár-Bereg) in Northeast Hungary, the area where the representation of Roma is the highest and where the majority of segregated Roma colonies are located. Firstly, 25 colonies with more than 100 inhabitants [37] were randomly selected; then, 20 households in each colony were randomly chosen, and one person aged 20–64 years from each household was interviewed face-to-face at the respondent’s household by Roma university students under the supervision of public health coordinators. The ethnicity of the participants was assessed by self-declaration [33].

The control study population involved randomly drawn individuals who were also between 20 and 64 years of age, lived in private households in the same counties of Northeast Hungary, and were registered by general practitioners (GPs). From 20 randomly selected GP practices, 25 individuals/practice selected at random were invited to participate in the study.

The planned sample sizes were 500–500 individuals for both study groups, but the final study sample, with full records of physical activity, included 797 participants: 410 subjects of the HG and 387 individuals of the HR populations.

### 2.2. Questionnaire-Based Interviews

The main part of the questionnaire in the complex survey was the European Health Interview Survey (EHIS) wave 2 (EHIS 2 for 2013–2015, used in the Hungarian survey in 2014) questionnaire [38] which consists of four modules on (a) health status, (b) health care use, (c) health determinants, and (d) socio-economic variables. The EHIS 2 questionnaire was extended with some additional groups of questions, among them the International Physical Activity Questionnaire (IPAQ) long-form to measure physical activity. IPAQ is designed to assess the time spent walking, doing moderate-intensity and vigorous-intensity activity according to different domains: (1) work, (2) transport, (3) domestic and garden, (4) leisure, and (5) time spent sitting in the last seven days. Data obtained from interviewer-assisted questionnaires were put into an Excel spreadsheet type database with the type of data as column headers and anonymized participants in the rows [33].

### 2.3. Analysis of Data on Physical Activity

In the analysis of physical activity, responses were converted to Metabolic Equivalent Task minutes per week (MET-min/week) according to the IPAQ scoring protocol [22]: total minutes over the last seven days spent on different types of physical activity to create MET scores for each activity category. MET scores across the several physical activity sub-components were analysed, and they were summed to indicate overall physical activity. According to the metabolic equivalent (MET) rates, three physical activity categories (low, moderate, and high) were defined. The items in the IPAQ long form were structured to provide separate domain-specific scores for walking, moderate-intensity, and vigorous-intensity activity within each of the work, transportation, housework and gardening, and leisure-time domains. The total scores for the long form are computed by the summation of duration (in minutes) and frequency (days) for all the types of activities in all domains. Domain-specific scores or activity-specific subscores could also be calculated by summation of the scores for walking, moderate-intensity, and vigorous-intensity activities within the specific domain, whereas activity-specific scores are the summation of the scores for the specific type of activity across domains [39,40].

Data collected with IPAQ were reported as a categorical variable as well.

These categories are:

1. Low physical activity (a minimum total physical activity of at least 600 MET-min/week, but less than 1500 MET-min/week);
2. Moderate physical activity (a minimum total physical activity of at least 1500 MET-min/week, but less than 3000 MET-min/week);
3. High physical activity (a minimum total physical activity of at least 3000 MET-min/week). All IPAQ data were cleaned and processed by Microsoft Excel Software using the standardised IPAQ Scoring Protocol [39]. The study population consisted of 797 participants (HG: 410 and HR: 387). As a first step, individuals with weekly physical activity greater than 6720 min (6720 min = 16 h × 7 days: There should be no more than 16 h of activity time because 8 h per day is the average sleep time according to the National Sleep Foundation [41]) were excluded (46 individuals). In the second step, 58 individuals were excluded because their record was not complete—693 individuals remained in the sample (HG = 343 and HR = 350).

### 2.4. Statistical Analysis

The frequency of physical activities was calculated by type in the dimensions of the different domains. A Pearson’s Chi-square test was used to compare prevalence data. The normality of data was tested by the Shapiro–Wilk test. For data with a non-normal distribution, the Mann–Whitney U test was applied. Values of *p* < 0.05 were considered to be statistically significant.

### 2.5. Ethical Statement

All subjects gave their informed consent for inclusion before they participated in the study. The study was conducted in accordance with the Declaration of Helsinki, and the protocol was approved by the Ethics Committee of the Hungarian Scientific Council on Health (61327-2017/EKU) [33].

## 3. Results

### 3.1. Socio-Demographic Characteristics of the Sample

A total of 797 adults participated in the study, and after data cleaning, 104 respondents were excluded (due to incorrect or incomplete data); finally, our sample consisted of 693 respondents, 350 HR and 343 HG people. Sixty-four per cent of the participants were female. They were overrepresented in both populations, and they had a significantly higher proportion in the HR (73.7%) than in the HG (55.2%) population (*p* < 0.001). There was no significant difference between the age distribution of the HG and HR populations. A significant difference (*p* < 0.001) was found between the two populations in terms of education, the HR population had a much lower level of education. As far as employment is concerned significant difference was also found (*p* < 0.001) (Table 1).

### 3.2. Physical Activity by Dimensions in the Study Samples

A higher proportion of HG men has low physical activity (22.02%) than HR men (17.21%), and significantly (*p* = 0.012) more HR men have moderate activity (51.62%) than HG men (42.14%), and more HR men have moderate activity (51.62%) than HG men (42.14%). Both differences are statistically significant (*p* = 0.011 and *p* = 0.012, respectively). Among women, a significantly higher proportion (*p* < 0.001) of HG females belong to the group of high activity (21.72%) than HR females (8.08%). Conversely, significantly (*p* < 0.001) more HR females have moderate activity (70.41%) than HG females (53.80%) Among women, a significantly higher proportion (*p* < 0.001) of HG females belong to the group of high activity (21.72%) than HR females (8.08%). Conversely, significantly more HR females have moderate activity (70.41%) than HG females (53.80%) (*p* < 0.001) (Figure S1).

Table 2 shows that although in certain domains the HR while in other ones the HG populations show greater levels of activity, these opposing differences cancel each other at the level of the weekly average.

### 3.3. Physical Activity by Domains in the Study Samples

Looking into the domains of physical activity (Figure S2), in both populations, male respondents achieve moderate or high levels of physical activity primarily through work with no significant difference between the two populations (HG: 44.54%, HR: 46.77%; *p* = 0.746) and secondarily through housework and gardening (HG: 32.59%, HR: 23.14%; *p* = 0.109). There was a significant difference in transport-related physical activity between HG and HR men. HR men (19.68%) move significantly (*p* = 0.025) more during transport than HG men (9.32%), i.e., they walk and cycle more. Transport-related physical activity also showed a significant difference (*p* = 0.030) among women in favour of HR women (23.52%) compared to HG ones (15.55%). Among women, physical activities of housework and gardening precede the level of work-related activity in both populations. Leisure time activity is the lowest among Roma women (9.54%), but the difference in comparison with HG women (14.06%) is not significant statistically (*p* = 0.169). Among men, the representation of leisure-time physical activity is also low both in HG and HR groups with no significant difference (13.55% and 10.42%, respectively, *p* = 0.547).

### 3.4. Physical Activity Dimensions in Different Domains

**Figure 1:**
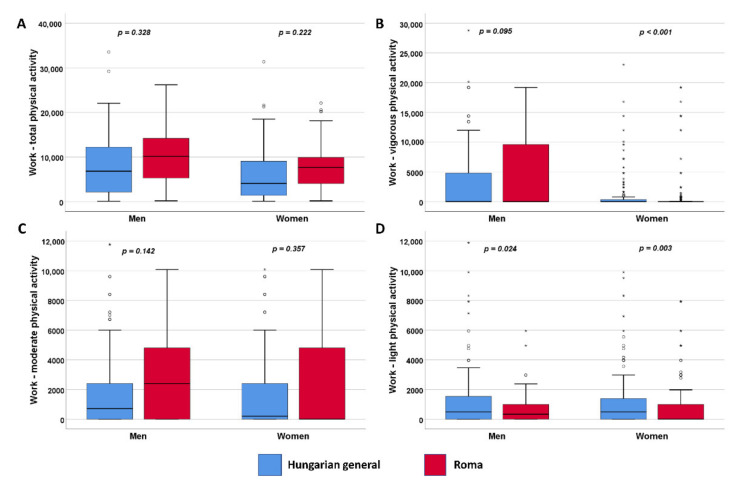
Distribution of physical activity in work (**A**) by intensity (**B**–**D**) expressed in METs. °: mild outlier data points; *: extreme outlier data points.

In the total work-related physical activity, there is no significant difference between the two populations, but there are significantly (*p* < 0.001) fewer HR females in the category of work-related vigorous physical activity. In both populations, most of the participants belong to moderate activity in the domain of work, and there are only a few people with low activity (Figure 1).

**Figure 2:**
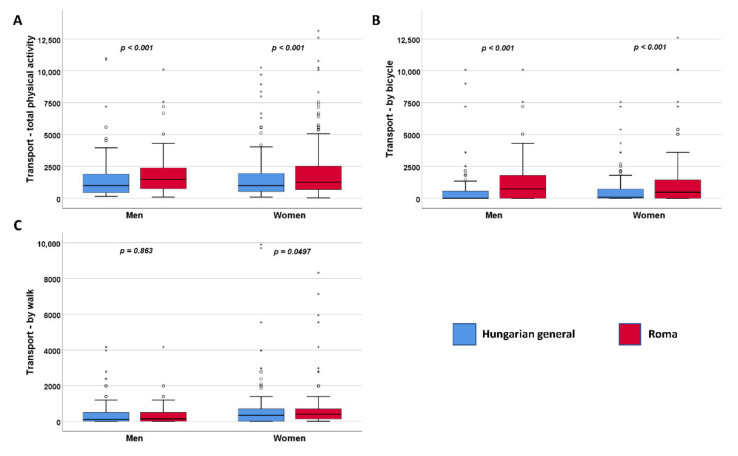
Distribution of physical activity in transport (**A**) by bicycle (**B**) and walk (**C**) expressed in METs. °: mild outlier data points; *: extreme outlier data points.

The HR population is significantly (*p* < 0.001) more active physically in transport, in comparison with the HG population. The reason for the difference is that both HR men and women are cycling significantly (*p* < 0.001) more (Figure 2).

**Figure 3:**
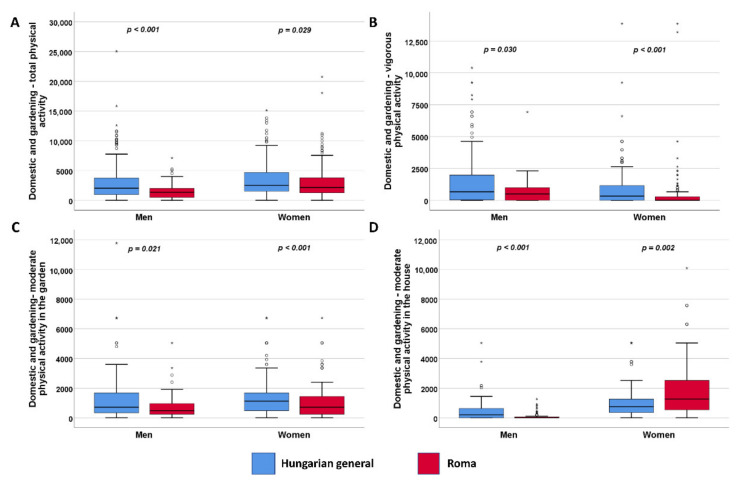
Distribution of physical activity in the domestic and gardening work domain (**A**) by intensity and setting (**B**–**D**) expressed in METs. °: mild outlier data points; *: extreme outlier data points.

In the domestic and gardening domains, the HG population shows more activity than the HR population. Regarding housework, HR women can be characterized by moderate activity in indoor housework, but HR men are active neither indoors nor outdoors (Figure 3).

**Figure 4:**
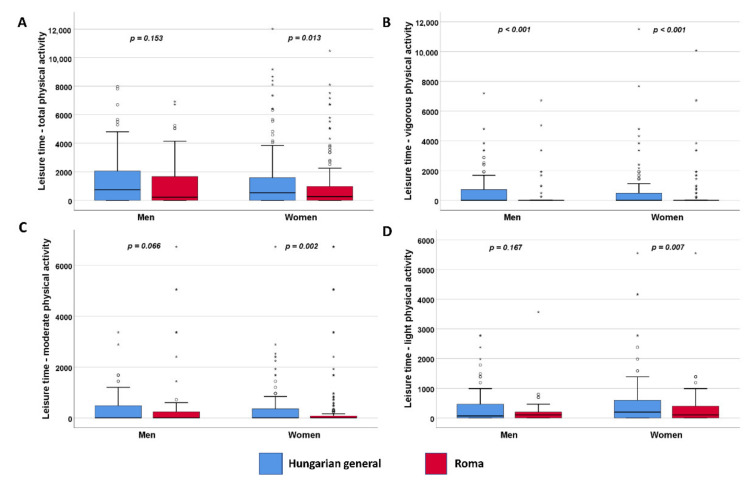
Distribution of leisure time physical activity (**A**) by types and intensity (**B**–**D**) expressed in MET. °: mild outlier data points; *: extreme outlier data points.

The leisure time activity of Hungarians is very low in general. In the present study, the prevalence of leisure time activity among both HG women and men was significantly (*p* <0.001) higher than among the HR population in the vigorous activity category. For women, this is also true for the moderate and light activity categories (*p* = 0.002 and *p* = 0.007, respectively) (Figure 4).

## 4. Discussion

We clearly show that the patterns of physical activity by dimensions differ significantly between the Roma and the general populations; namely, for Roma in both sexes, moderate physical activity is more characteristic. In the category of work-related vigorous physical activity, there are significantly fewer females among Roma than in the general population. By domains, male respondents achieve moderate or high levels of physical activity primarily through work with no significant difference between the two populations, but in both sexes, a significant difference could be observed in transport-related physical activity in favour of the Roma. Leisure-time physical activity is very low among Roma in both sexes (the lowest among Roma women), but the differences between HR and HG groups are not significant statistically, because it is severely insufficient also in the general population.

In a pooled analysis of 358 population-based surveys with 1.9 million participants it is showed that the age-standardised world prevalence of insufficient physical activity has been stable since 2001, and it was 27.5% (with 95% uncertainty interval 25.0–32.2) in 2016. The prevalence of insufficient physical activity varied in a wide range (5.5–67%), and in most of the European countries, it is over 30% [42].

In the only study which previously examined the physical activity of Roma in a survey of epidemiologically correct design [40], the method used to define dimensions was described as “physical activity was measured by asking respondents two questions on what physical activity they had done during the last week and how often they performed physical activity lasting at least 30 min, during which they became breathless or sweaty. Those who reported being physically active 2 or more times a week were considered to be sufficiently physically active.” Although they tried to characterize physical activity by domains, their findings strongly differ from ours: physical activity at work was described as significantly lower among Roma for both sexes than in the Slovakian general population (it was reported by 21.4% of men and 18.1% of women living in Roma settlements, but by 41.6% of men and 25.2% of women from the majority population), while significantly higher activity indoor and outdoor around the house was reported by more female respondents living in Roma settlements in comparison with the majority population (82.6% vs. 67.9%). In contrast, in our study, we found no significant difference between the two populations in total work-related physical activity, and total physical activity in the domestic and gardening domain was found significantly lower for both sexes in our sample. We could show also for both sexes among Roma that transport-related physical activity was significantly higher than that in the general population, which is based on the fact that the Roma use bicycles for transport.

Regarding the leisure-time physical activity findings of an Ipsos online survey conducted in 29 countries (12 European countries among them) with an international sample of 21,503 adults aged 16–74 in 2021, it shows very variable data for different countries [43]. Just four per cent of people from the Netherlands say that they do not exercise at all in a normal week, while in Italy and Poland, more than a quarter of the people say the same. Men in the Netherlands and women in Germany are the most active (average mean numbers of hours per week they spend doing physical exercise are 15.2 and 11.3, respectively), while French men and Italian women report the lowest average time spent doing physical exercise per week (mean numbers of hours are 4.2 and 2.7, respectively). Regarding sporting activities, only 13% of Hungarians and Italians practise fitness (the lowest proportion among European countries), and only 5% of Hungarians play soccer in a normal week. Citizens of Great Britain and Italy are the most likely to say they do not play any team sports in a normal week (59% and 53%, respectively), but in most of the European countries, more than 50% of the survey participants say that they would like to practice more sport than they currently do. Barriers (such as lack of time and/or money, lack of facilities, etc.) that stop them from practising sports as much as they would like are identified by country, but quite a high proportion of people say that there are no barriers to their participation in sport, but they simply do not want to play/take part. Considering that cycling is not only a type of sporting activity but also a form of transportation, the 6–30% representation of people ever cycling in a normal week in the European countries is considered low.

In addition, data from Eurobarometer 2014 and 2018 show that the western European population shows a higher tendency to do physical activity than Eastern Europeans, and northerners are, actually, more active than southerners [44,45]. The situation is especially alarming in Hungary, Romania, and Turkey, where none of the age groups above 20 reported their participation in physical activity to exceed 10%. Their rates largely depend on the income situation of the region, as well as the prevalent social and cultural views. It is assumed that in places where the desire to increase sport activity is hindered by low income, it is extremely difficult to produce changes [46]. However, low-level physical activity results in a decline in people’s health and an increase in health expenditure. Consequently, conducting health economic studies in the region gains substantial importance [47].

The findings of the Slovakian study and ours on leisure-time physical activity are partly compressed—total activity was significantly lower only for women among Roma, but in the vigorous dimension, it was significantly lower also for men.

Regarding the total physical activity of the HR population, our research findings partly contrast also with the results of Szabó et. al. [48]. They reported different results for Roma people living in Romania when they compared the physical activity patterns of two different Roma populations (Gabor Roma N = 231 and Băieși Roma *n* = 111) to that of the non-Roma population (N = 183). In contrast with our results, physical activity was found to be lower by them in both Roma groups than in the case of the non-Roma population, but in harmony with our findings, the representations of gardening and leisure time activities were found to be much lower for both Roma groups as compared to the non-Roma.

Fónai et al. [49] studied 500 Roma households about the subjective health indicators of the Roma in Northeast Hungary. In addition to finding a high rate of various addictions among the Roma population, they confirmed the disadvantaged socioeconomic background of the Roma. Most of them lived below the breadline, below the relative poverty threshold that was accompanied by a low level of education and a high unemployment rate.

The findings of Vokó et al. [30] proved that the health conditions of those living in Roma-inhabited colonies are poorer than those of the average population. This study has established that to improve the health conditions of the Roma, we need to focus on cultural differences too, in addition to their socio-economic situation in general. This corresponds to our finding that certain characteristics—in our research certain habits related to physical activity, such as the fact that Roma people typically prefer walking as the primary means of commuting and bicycling for transportation—can predict Roma ethnicity.

Hüse and Pénzes [31] investigated the health-related behaviour of 154 Roma adults. They have found that 50% of the subjects in their sample had never done any kind of sports before, but 46.86% of the participants claimed to do physical activity that made them sweat several times a week. This corresponds to our finding that Roma people are characterized by moderate or high physical activity in terms of work and homework.

Ember [50] conducted a focus-group study on health-related behaviour and quality of life among young Roma women (19–29 years of age) living in Nyíregyháza, the county capital of Szabolcs-Szatmár-Bereg, in slums. Just like this study, the research of Ember also provided evidence for Roma women’s activity in household chores, as well as for their reluctance to do sports. Young women involved in the study were mostly mothers, who explained their reluctance to do sports with their motherhood responsibilities and household chores. When asked about men, respondents replied that they were doing physical work to ensure the livelihood of the family, and after such activity, they did not intend to do sports. Such findings correspond to our results.

In a case study in Hungary in the framework of the project of BAGázs Public Benefit Association in 2011 in Bag, a settlement with a Roma colony of about 400 Roma inhabitants, the social integration and involvement of the Roma minority was targeted through sports. Their interventions were carried out at the micro-level in Roma slums, at meso-level among local village communities, as well as at the macro-level targeting the social sensitization of the majority of society. By providing sports classes, the program aimed to achieve the socio-economic integration of the Roma in Bag. Volunteers coached Roma people in football, where the primary aim was—instead of improvement of sports performance—to develop positive personal qualities such as discipline, willpower, persistence, and team spirit. Based on the achievements of the program, the association fighting against extreme poverty and segregation has concluded that one obstacle to the Roma integration is the inability-focused approach towards their fate, which characterizes Roma people living in the slums. Such approach is attributed to inadequate socialization, poor communication patterns, and lack of basic moral values, as well as to low education, early school leave, high unemployment rate, and early childbearing as a consequence of the above. Another cause is social prejudice. Several studies have pointed out that sports can help inclusion and social integration [51,52,53].

Sanz-Remacha et al. [54] classified the hindrance factors of physical activity into three categories: personal (economic, work-related, physical obstacles, illness, and psychological features), social (culture, lack of social support, and family), and environmental. Roma people involved in our study sample are supposed to be affected by all three categories. The following factors have been identified: in the case of the personal category, relatively poor socio-economic background (lower education, lower employment rate); in terms of cultural determination, Roma habits (e.g., avoid doing sports after work, commute by walk); and in the case of decisive environmental conditions, village lifestyle without own land [54].

The major limitation of the survey from which the data on physical activity were obtained is that females are overrepresented in the Roma sample. As we described previously [54], this complex health survey was based on randomly selected households, and in many households, only women were home during the day when most visits took place, while men were away for public work. The Hungarian government quadrupled the budget for public works between 2010 and 2015 for all municipalities, which most strongly affected villages in the North-eastern region of Hungary, where segregated Roma settlements are concentrated.

## 5. Conclusions

Our study focuses on the physical activity of the Roma population in comparison with the general population in Hungary. The Roma and the general population in Hungary are both characterized by a high level of activity at work and housework. For Roma people, however, heavy physical work is more typical, while in housework and gardening the Hungarian general population shows higher activity. Our hypothesis that Roma people are characterized by lower physical activity compared to the majority of people in society due to their socioeconomic disadvantages has not been substantiated. It has not been proven that household and garden activity dominate in their physical activity, but it has been proven that due to walking and cycling, their transport-related physical activity is higher than that of Hungarians.

In terms of leisure time activities, an enormous difference has been found, and in such respect, the Roma lag significantly behind the majority of society.

The results also highlight the role of sports in the health preservation and social integration of the Roma. Leisure time sports programs should be organized for all age groups of the Roma population in each settlement. Play competitions are recommended for children, musical and dance movements for women, and ball games for men. The presented example of Bag—where Roma people have been offered sports activities under the framework of volunteer work—proves the cost-efficient nature of such practice. Our concluding suggestion is that both populations should increase the activity of recreational sports, and the Hungarian population can also develop its transport-related activity by walking and cycling more. It is important to note that in the light of results obtained in a high number of studies mainly in the past two decades, physical activity and sedentary behaviour are considered independent research fields [55]. As emphasized by Torres et al. [55], it seems crucial to find strategies not only to increase physical activity but to reduce sedentary behaviour in all life domains. Including regular and well-structured sedentary breaks during long sitting periods at working places could help reduce the negative effects of a sedentary lifestyle.

## Figures and Tables

**Table 1:** Socio-demographic characteristics of the sample populations by sex.

		Men			Women		
		Hungarian General (N = 148)	Roma (N = 92)	*p*-Value	Hungarian General (N = 195)	Roma (N = 258)	*p*-Value
		(95% CI)			(95% CI)		
Average age (years)		44.6 (42.7–46.5)	44.9 (42.2–47.7)	0.589	43.7 (41.8–45.5)	42.2 (40.7–43.6)	0.186
Age groups’ distribution	20–34-year-old	20.9% (15.0–28.0)	26.1% (18.0–35.7)	0.114	30.8% (24.6–37.5)	29.5% (24.1–35.2)	0.084
	35–49-year-old	43.9% (36.1–52.0)	30.4% (21.7–40.3)		29.7% (23.7–36.4)	39.1% (33.3–45.2)	
	50–64-year-old	35.1% (27.8–43.1)	43.5% (33.7–53.7)		39.5% (32.8–46.5)	31.4% (26.0–37.2)	
Distribution by education categories	Less than primary and primary	18.2% (12.7–25.0)	88.0% (80.3–93.5)	<0.001	22.1% (16.7–28.3)	85.7% (81.0–89.5)	<0.001
	Vocational and high school	63.5% (55.6–70.9)	12.0% (6.5–19.7)		59.0% (52.0–65.7)	14.0% (10.1–18.6)	
	College and university	18.2% (12.7–25.0)	---		19.0% (13.9–24.9)	0.4% (0.0–1.8)	
Distribution by employment categories	Missing	2.0% (0.6–5.3)	1.1% (0.1–5.0)	<0.001	1.5% (0.4–4.0)	0.8% (0.2–2.5)	<0.001
	Employed	78.4% (71.2–84.4)	40.2% (30.6–50.4)		68.7% (62.0–74.9)	45.0% (39.0–51.1)	
	Casual worker or unemployed	9.5% (5.5–15.0)	42.4% (32.7–52.6)		2.6% (1.0–5.5)	28.3% (23.1–34.0)	
	Retired or disabled	8.1% (4.5–13.3)	14.1% (8.2–22.3)		13.8% (9.5–19.2)	8.9% (5.9–12.9)	
	Other not employed	2.0% (0.6–5.3)	2.2% (0.5–6.8)		13.3% (9.1–18.6)	17.1% (12.8–22.0)	

**Table 2:** Physical activity expressed in average METs per week by intensity categories among men and women in the Hungarian general and Roma populations.

	Men			Women		
	Hungarian General (N = 148)	Roma (N = 92)	*p*-Value	Hungarian General (N = 195)	Roma (N = 258)	*p*-Value
	Average (95%CI)			Average (95%CI)		
Vigorous	5145.69 (4167.41–6123.97)	5307.42 (3898.63–6716.20)	0.195	2648.01 (2099.38–3196.65)	1277.59 (836.62–1718.55)	<0.001
Moderate	4567.36 (3924.87–5209.86)	5495.65 (4467.72–6523.59)	0.306	4954.08 (4440.86–5467.29)	6545.27 (5898.61–7191.93)	0.009
Light	2186.58 (1797.39–2575.78)	1238.29 (938.62–1537.96)	0.001	2225.64 (1894.71–2556.57)	1698.73 (1451.97–1945.50)	0.011
Total physical activity	11,899.64 (10489.83–13309.45)	12,041.36 (10208.42–13874.30)	0.981	9827.73 (8840.73–10814.73)	9521.59 (8636.33–10,406.85)	0.469

## Data Availability

Data are available on request due to privacy or ethical concerns.

## Supplementary Materials

The following are available online at https://www.mdpi.com/article/10.3390/ijerph19063545/s1, Figure S1: Representation of intensity categories (vigorous, moderate, and light) of physical activity in the study populations among men (A) and women (B), Figure S2: Representation of different domains (work, transport, domestic and garden, and leisure-time) of physical activity in the study populations among men (A) and women (B).
