# Supplementary material for: Physical Activity Pattern Characterized by Domains and Dimensions of the Roma Population in Comparison with That of the General Population in Northeast Hungary
Source: Int J Environ Res Public Health. 2022 Mar 16;19(6):3545. doi: 10.3390/ijerph19063545 (PMC8948787; doi:10.3390/ijerph19063545)
Supplement: Supplementary file 1 [file ijerph-19-03545-s001.zip › ijerph-1621650-supplementary.pdf]

Supplements

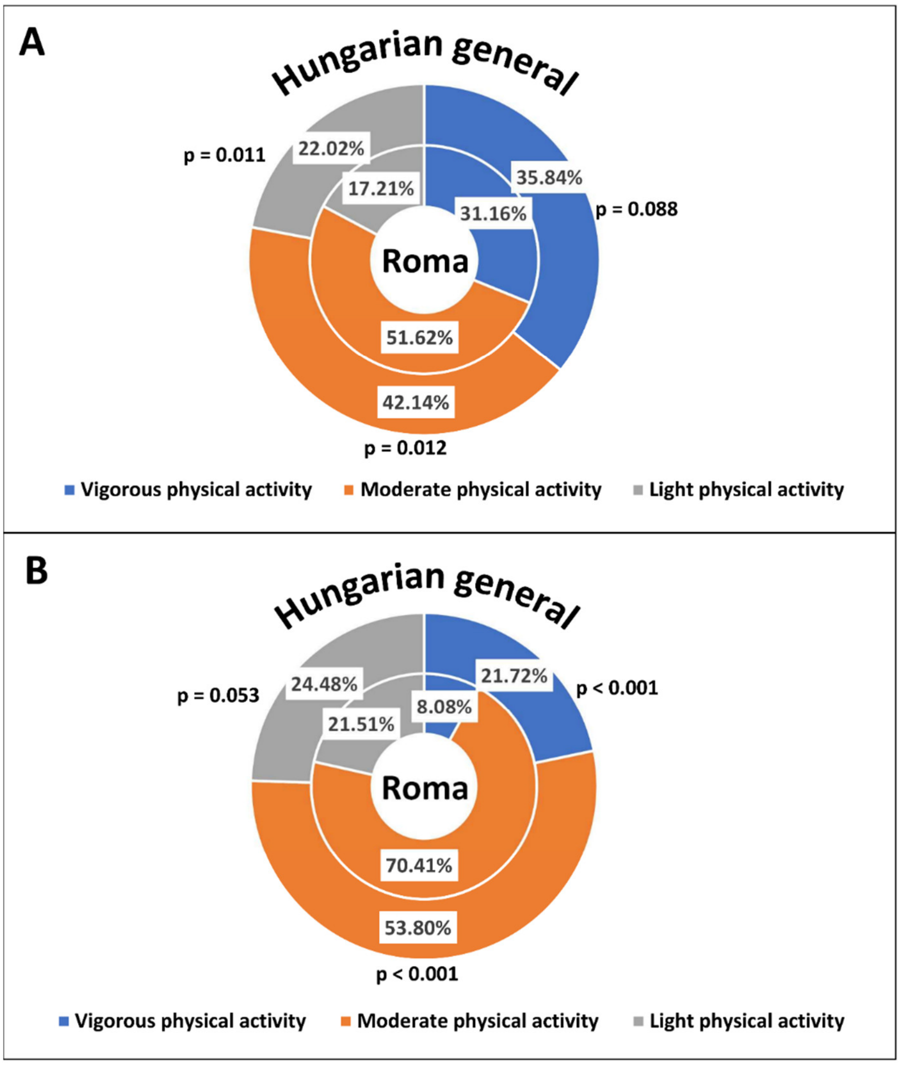

Figure S1. Representation of intensity categories (vigorous, moderate, and light) of physical activity in the study populations among men (A) and women (B).

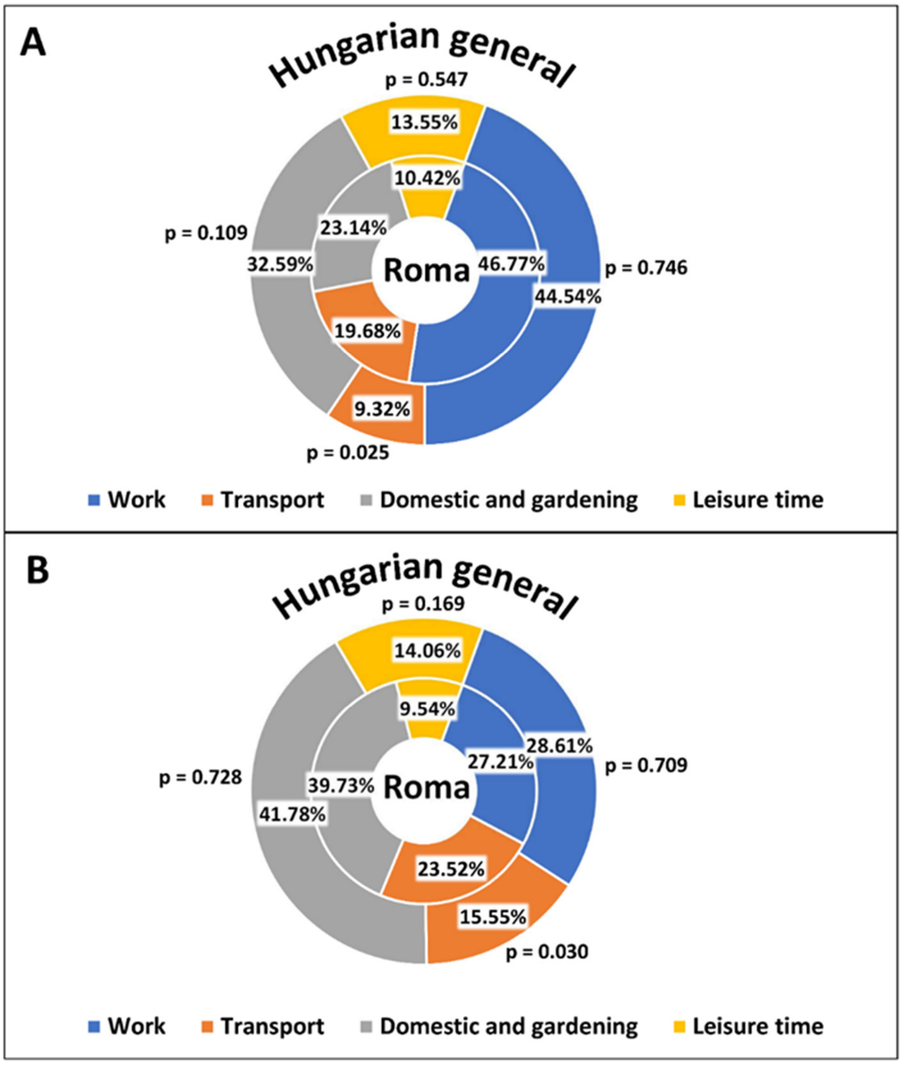

Figure S2. Representation of different domains (work, transport, domestic and garden, and leisure-time) of physical activity in the study populations among men (A) and women (B).
